# Adaptive Evolution of the *Hox* Gene Family for Development in Bats and Dolphins

**DOI:** 10.1371/journal.pone.0065944

**Published:** 2013-06-25

**Authors:** Lu Liang, Yong-Yi Shen, Xiao-Wei Pan, Tai-Cheng Zhou, Chao Yang, David M. Irwin, Ya-Ping Zhang

**Affiliations:** 1 State Key Laboratory of Genetic Resources and Evolution, Kunming Institute of Zoology, Chinese Academy of Sciences, Kunming, China; 2 State Key Laboratory of Cellular Stress Biology, School of Life Sciences, Xiamen University, Xiamen, China; 3 Graduate School of the Chinese Academy of Sciences, Beijing, China; 4 Laboratory for Conservation and Utilization of Bio-resources, Yunnan University, Kunming, China; 5 Department of Laboratory Medicine and Pathobiology, University of Toronto, Toronto, ON, Canada; 6 Banting and Best Diabetes Centre, University of Toronto, Toronto, ON, Canada; Sars International Centre for Marine Molecular Biology, Norway

## Abstract

Bats and cetaceans (i.e., whales, dolphins, porpoises) are two kinds of mammals with unique locomotive styles and occupy novel niches. Bats are the only mammals capable of sustained flight in the sky, while cetaceans have returned to the aquatic environment and are specialized for swimming. Associated with these novel adaptations to their environment, various development changes have occurred to their body plans and associated structures. Given the importance of *Hox* genes in many aspects of embryonic development, we conducted an analysis of the coding regions of all *Hox* gene family members from bats (represented by *Pteropus vampyrus*, *Pteropus alecto*, *Myotis lucifugus* and *Myotis davidii*) and cetaceans (represented by *Tursiops truncatus*) for adaptive evolution using the available draft genome sequences. Differences in the selective pressures acting on many *Hox* genes in bats and cetaceans were found compared to other mammals. Positive selection, however, was not found to act on any of the *Hox* genes in the common ancestor of bats and only upon *Hoxb9* in cetaceans. PCR amplification data from additional bat and cetacean species, and application of the branch-site test 2, showed that the *Hoxb2* gene within bats had significant evidence of positive selection. Thus, our study, with genomic and newly sequenced *Hox* genes, identifies two candidate *Hox* genes that may be closely linked with developmental changes in bats and cetaceans, such as those associated with the pancreatic, neuronal, thymus shape and forelimb. In addition, the difference in our results from the genome-wide scan and newly sequenced data reveals that great care must be taken in interpreting results from draft genome data from a limited number of species, and deep genetic sampling of a particular clade is a powerful tool for generating complementary data to address this limitation.

## Introduction

Chiroptera and Cetacea have undergone adaptive radiation with their specialized lifestyles during the Cretaceous Terrestrial Revolution and the Cretaceous-Paleogene mass extinction events [1]. Bats are unique mammals, which occupy the aerial niche [2]. From a terrestrial ancestor, several physiological and morphological changes were required by bats to acquire the ability of flight. Adaptive evolution of mitochondrion-associated genes has been shown in a previous study to play a critical role in the origin of flight in bats [3]. In addition to an increase in energy demand, flight also requires a large amount of change in their external morphology. One remarkable character is the structural evolution of wings from forelimbs, as this made flapping flight possible [4]. In the bat wing, the phalanges have become extremely elongated (especially the third, fourth, and fifth digits) to brace the stretched membrane [5], with flight muscles, such as pectoralis muscle, being the ‘engines’ that support flight. Flight muscles must produce sufficient power for flight, thus all muscle fibers of bats are adapted for fast-twitch contractile capability, highly oxidative, and poorly suited for glycolytic (anaerobic) metabolism [6], [7], [8], [9]. Other innovations, such as having a robust lung with high-performance blood-gas exchange were also essential for the attainment of flight [10]. Cetaceans are a second group of exceptional mammals, which are commonly known as the whales, dolphins, and porpoises. From a terrestrial ancestor, cetaceans re-entered the sea and re-acquired an aquatic lifestyle by at least the mid-Eocene [11]. A streamlined body shapes helps them move freely in the aquatic environment by reducing the frictional resistance from water molecules. A pair of paddle-shaped fore-flippers in cetaceans is equivalent to the forelimbs of typical land mammals, where skeletal changes, including number of bones and pattern, formed the narrow and elongated flippers which facilitate the dispersion edge forces that allow fast swimming [12], [13]. In contrast to the highly developed forelimbs, the hindlimbs of cetaceans are virtually absent. Locomotion in cetaceans is accomplished by the vertical movement of their tails [14]. Adaptation of the skin in cetaceans is characterized by their lack of glands and hair [14], while their thick blubber, and countercurrent heat exchange systems, help them cope with the cold [15]. Similarly to bats, the strong muscular system has evolved in association with their life in an aquatic environment [14]. Both bats and cetaceans, therefore, have developed specialized body plans and associated physiological systems to allow them to adapt to new lifestyles during their evolution from terrestrial ancestors.

*Hox* genes encode transcription factors that regulate the level of expression of many downstream target genes to control the primary and secondary axes during development [16], [17], [18]. Vertebrates usually have four distinct *Hox* gene clusters (*Hox A, B, C,* and *D*), which are located on different chromosomes [19]. Among the clusters, paralogs are arranged in a collinear manner [20]. Each paralog is successively activated, from ‘head’ to ‘tail’, and makes contributions throughout embryonic development [21]. In vertebrates, *Hox* genes have a direct role in controlling cellular movement during gastrulation, thereby contributing to body formation [22]. In addition, members of the *Hox* genes are required to be expressed in the mesoderm to promote the proliferation and differentiation of skeletal progenitor cells, and the recruitment of mesenchymal cells into precartilaginous condensations during limb development [23], [24]. Several *Hox* genes contribute to the development of hair follicles [25] and *Hoxc13* mutant mice lack external hair [26]. *Hoxc8* functions in the pathway that determines white and brown adipose tissues [27]. The expression of three ‘anterior’ *Hox* genes (*Hoxa1*, *Hoxa2* and *Hoxa3*) is correlated with specific portions of the hindbrain [28]. Recently it was shown that regulatory interaction between *Hox* members (*Hoxb1* and *Hoxb2*) is involved in sound perception through their contribution to the assembly of rhombomere elements [29]. *Hox* genes, therefore, make broad contributions to development, including those that have adapted in bats and cetaceans. Previous studies were focused on the expression pattern of *Hox* genes, as variation in the spatio-temporal pattern of expression of *Hox* proteins correlated with morphological differences [16]. For example, comparison of expression pattern of the *Hoxd11* gene between mouse and zebrafish reveals that the axis of expression curves in the mouse while the axis in the pectoral fin of the zebrafish remains straight, although the early expression pattern is same in both species [30]. *Hox*-protein-function is also important for *Hox* function [16], thus mutations in *Hox* protein sequence also affect morphological evolution. For example, genes in the *HoxA* and *HoxD* clusters (*Hoxa9*–*Hoxa13* and *Hoxd9*–*Hoxd13*) are particularly important in vertebrate limb development [28]. Limb malformations, e.g., synpolydactyly and hand-foot-genital syndrome in humans, are caused by mutations in *HOXD13* and *HOXA13*, respectively [31]. Missense mutations in *HOXD4* have been found in childhood cases of acute lymphoid malignancy [32]. Thus, *Hox* genes are widely recognized as candidates for controlling morphological diversity by changing expression pattern and protein sequence.

Here, we globally examined the sequence of *Hox* gene family members during the adaptive evolution on new lifestyles in bats and dolphin. To complement and validate these results from the genomic surveys, we newly sequenced part of the *Hox* genes from additional bat and cetacean species. Analyses of these data reveal that *Hoxb2* in bats and *Hoxb9* in cetaceans show significant positive selection, while most of the other *Hox* genes evolve conservatively. Given the contradiction between conserved *Hox* sequences and specialized morphology in both bats and dolphin, we speculate that changes in labile *Hox*-expression-patterns likely make a greater contribution to the development in bats and dolphin, rather than changes in *Hox*-protein-function, either through variation in the expression pattern of *Hox* itself or through alterations of its activation of downstream target gene promoters.

## Materials and Methods

### Primary data from genomes

*Hox* Genes from the four distinct clusters (*Hox A, B, C,* and *D*) were studied in this analysis. These genes were: *Hoxa1*, 2, 3, 4, 5, 6, 7, 9, 10, 11, 13; Hoxb1, 2, 3, 4, 5, 6, 7, 8, 9, 13; Hoxc4, 5, 6, 8, 9, 10, 11, 12, 13; Hoxd1, 3, 4, 8, 9, 10, 11,12, 13. All of the human (*Homo sapiens*) Hox genes were collected from the Ensembl public database (release 64), then using the Ensembl ortholog_one2one gene database, we obtained the corresponding orthologs from the ﬂying fox (*Pteropus vampyrus*), little brown bat (*Myotis lucifugus*), dolphin (*Tursiops truncatus*), macaque (*Macaca mulatta*), chimpanzee (Pan troglodytes), orangutan (*Pongo pygmaeus*), mouse (*Mus musculus*), rat (*Rattus norvegicus*), dog (*Canis familiaris*), horse (*Equus caballus*), and cow (*Bos taurus*). In addition, we added two other bat species (*Pteropus alecto* and *Myotis davidii*) with newly published genome data [33]. Since the two new bat genomes lacked annotation, the individual *Hox* genes in *P. alecto* and *M. davidii* were identified and confirmed using reciprocal sequence searches and alignments using orthologous regions from the other mammals. Putative coding regions were then identified using the GeneWise program (http://www.ebi.ac.uk/Tools/psa/genewise/).

### Amplification and sequencing of*Hox* genes from deep genetic samplings

DNA was extracted from 23 species of bats distributed across six families and thirteen genera, representing their phylogenetic diversity as well as two species of cetaceans (see Table S1 for list of species). PCR was used to attempt to amplify both exons from all 39 *Hox* genes in all 23 species of bats and in the two cetaceans. Degenerate primers for each exon of the *Hox* genes were designed to conserved flanking regions based on the aligned sequences from the species collected from Ensembl described above. Regions showing variable amino acid sequence, based on the comparison of genomic sequences, were also amplified. PCR primers for successful amplification are listed in Table S2. Total genomic DNA was extracted using the standard 3-step phenol/chloroform extraction method [34]. PCR amplifications were carried out using the following program: 2 min at 95°C, 10 cycles of 1 min at 94°C denaturation, annealing at 60–50°C (30 sec; −1.0°C/cycle), extension for 1 min at 72°C, followed by 25 cycles of 1 min at 94°C, 30 sec at 50°C, and 1 min at 72°C. PCR products were purified using Watson PCR Purification Kits (Watson BioTechnologies, Shanghai), and sequenced bidirectionally on an ABI 3730 Sequencer (Applied Biosystems, Foster, CA, USA) using ABI PRISM BigDye Terminator v3.0. Raw sequences were edited using DNAstar Seqman software (DNASTAR Inc., Madison, WI, USA). Newly determined sequences were deposited into GenBank (Accession numbers JN013209-JN013929, also shown in Table S2; with a few short exon sequences of less than 200 bp given in Table S3, and could not be deposited into GenBank due to their short length). All the sequences were aligned using ClustalX 1.81 [35] and visually checked for accuracy.

### Molecular evolutionary analyses

**Figure 1:**
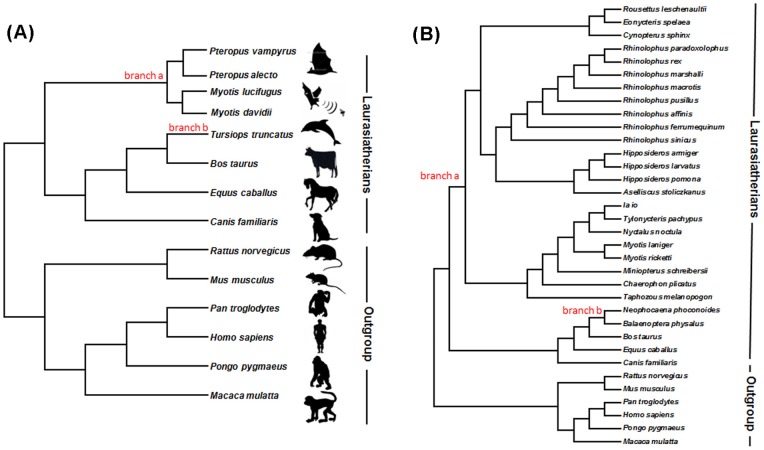
Phylogenetic tree of the species used for the evolutionary analysis of*Hox* genes. Phylogenetic trees were from previous analyses [2], [37], [38]. (A) Species tree for the analyses of the genomic data. (B) Species tree for the analyses of the newly sequencing data.

We used the CODEML program in PAML 4 [36] to estimate the rates of synonymous (*dS*) and nonsynonymous substitutions (*dN*) and the *dN/dS* ratio (omega, ω). *dN/dS* >1 suggests positive selection, *dN/dS* <1 indicates negative selection, and *dN/dS*  = 1 is neutrality. The species trees (Figure 1(A) and 1(B)), which were used as guide trees for the genome data and directed sequencing data analyses, respectively, were based on previous studies [2], [37], [38]. For all PAML-based analyses, alignment gaps were treated as ambiguous characters. Branch lengths were estimated simultaneously (iteration setting method  = 0), and codon frequencies were calculated from the average nucleotide frequencies at the third codon positions (setting CodonFreq  = 2 (F3X4)).

Initially, we used the one-ratio model (M0), a very strict model, which allows only a single *dN/dS* ratio for all branches to estimate the general selective pressure acting among all species. Free-ratios model (M1) was then used to analyze the *dN/dS* ratio along each branch. Branch models, which allow the *dN/dS* ratio to vary only among branches, were subsequently conducted to compare the selective pressure between bats or dolphin and other mammals. To further examine potential positive selection acting on the *Hox* genes along the branch leading to the common ancestor of all bats and the branch leading to cetaceans, the branch-site test of positive selection (test 2), which allow variation of the *dN/dS* ratio to occur at both amino acid sites and on lineages, was used as it generates lower rates of false positive results [39]. For the branch models and branch-site test 2, branches leading to the ancestor of the bats (branch a in Figure 1) and to the cetaceans (branch b in Figure 1) were chosen as the foreground branches separately. Statistical significance was assessed using LRTs (likelihood ratio tests).

### Phylogenetic analyses

For the maximum likelihood (ML) analysis, the model in the amino acid sequences was chosen by the Akaike information criterion (AIC [40], [41]) using ProtTest 3.0 beta [42] and a ML tree was reconstructed with RAxML [43]. Bootstrap re-sampling was used to assess support for the nodes on the tree using 100 replicates. Sequences of the human and mouse genes were chosen as references for the analysis of our sequencing data.

## Results

### Survey for positive selection in genomic data

All human *Hox* genes (39 genes) were acquired from the Ensembl database and their orthologues from 11 mammals were identified using the Ensembl ortholog_one2one tool. Coding regions of *Hox* genes from *P. alecto* and *M. davidii* were predicted from genomic contigs using GeneWise. If multiple transcriptional forms for a gene were annotated, the longest transcript was chosen. Some genes are missing in some genomes, likely due to gaps in the genome assemblies, thus our final analysis included 39 *Hox* genes from bats and 33 *Hox* genes from the dolphin (*Hoxa9, Hoxa13, Hoxb7, Hoxc11, Hoxd10,* and *Hoxd11* were missing). The Ensembl transcript ID and GenBank accession numbers of all *Hox* genes used in our study are listed in Table S4. The annotated positions of each exon for the *P. alecto* and *M. davidii Hox* genes are also listed in Table S4.

**Figure 2:**
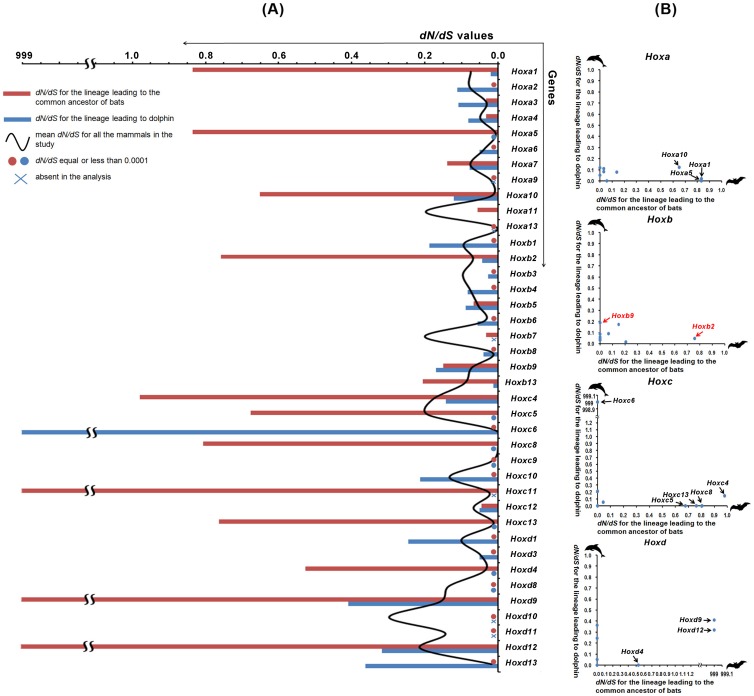
*dN/dS* ratios are heterogeneous across the *Hox* genes. (A) The mean *dN/dS* values are from the M0 model, while the *dN/dS* values for the bat ancestor and the dolphin are from the free ratio model (Model M1). (B) Differences in the *dN/dS* values between bats and dolphin. Data are from the M1 model.

We assessed the global evolutionary pressures acting on the *Hox* genes in bats and dolphin by comparing their *dN/dS* values (data from Model M1) with the mean values from all mammals (data from Model M0) for each *Hox* gene separately. The average *dN/dS* values for each *Hox* gene in our investigated mammals range from 0.0001 to 0.2988 (Table S5). As shown in Figure 2(A), the *dN/dS* values of some bat and dolphin *Hox* genes are comparatively larger than the corresponding average values of other mammals. Interestingly, within the *Hox* gene clusters, some genes showed opposite evolutionary pattern between bats and dolphin (Figure 2(B)), including three genes, *Hoxa1* (ω_bat_: 0.8350, ω_dolphin_: 0.0194, ω_average_: 0.0749), *Hoxa5* (ω_bat_: 0.8348, ω_dolphin_: 0.0001, ω_average_: 0.0063), *Hoxa10* (ω_bat_: 0.6516, ω_dolphin_: 0.1202, ω_average_: 0.0208) in the *HoxA* cluster; one gene, *Hoxb2* (ω_bat_: 0.7583, ω_dolphin_: 0.0443, ω_average_: 0.0687) in the _HoxB_ cluster; five genes, *Hoxc4* (ω_bat_: 0.9799, ω_dolphin_: 0.1420, ω_average_: 0.1785), *Hoxc5* (ω_bat_: 0.6762, ω_dolphin_: 0.0001, ω_average_: 0.1925), *Hoxc6* (ω_bat_: 0.0001, ω_dolphin_: 999, ω_average_: 0.0079), *Hoxc8* (ω_bat_: 0.8057, ω_dolphin_: 0.0001, ω_average_: 0.0001), *HoxC13* (ω_bat_: 0.7626, ω_dolphin_: 0.0001, ω_average_: 0.0126) in the *HoxC* cluster; and three genes, *Hoxd4* (ω_bat_: 0.5274, ω_dolphin_: 0.0001, ω_average_: 0.0363), *Hoxd9* (ω_bat_: 999, ω_dolphin_: 0.4104, ω_average_: 0.1595), *Hoxd12* (ω_bat_: 999, ω_dolphin_: 0.3182, ω_average_: 0.2127) in the *HoxD* cluster.

We next analyzed each *Hox* gene in the bats and the dolphin with more rigorous models, including the branch models and the branch-site models test 2. The monophyly of bats is unambiguously supported both by morphology and molecular data [37], [44], [45], thus their common ancestor, namely branch a (Figure 1 (A)) was selected as the foreground branch for our study. The selective pressure acting on each of the *Hox* genes on this branch was subsequently calculated, however, both the branch and the branch-site models failed to detect any evidence for positive selection acting on any of the genes along this branch leading to bats. The result of this analysis is shown in Table S5.

The same type of analysis was used to assess the selective pressures acting on the *Hox* genes along the dolphin branch (branch b, in Figure 1 (A)). Branch models again failed to detect any signals of positive selection, however, when branch-site models (test 2) were applied, *Hoxb9* was suggested to have undergone positive selection (LRT test, 2⊿l = 3.8683, df = 1, P<0.05) with one site (175 P) having a BEB value (posterior probability) >0.99 (Table 1).

**Table 1:** The parameters of selective pressure for*Hoxb9* in dolphin from genomic data.

Model	Inl			Estimates of parameters			
**MD**	−1471.717609	Ω = 0.0729					
**MI**	−1460.844369	ω_dolphin_ = 0.1706					
**Branch Models**							
**Dolphin**	−1470.859301	ω_dolphin_ = 0.1722	Ω_0_ = 0.0654				
**Dolphinω_dolphin_**	−1474.048375	Ω_0_ = 0.0654					
**Branch-Site Test 2**							
**Dolphin**	−1467.388728	Site class	0	1	2a	2b	Positively selected site
		proportion	0.98549	0.00523	0.00923	0.00005	175 P 0.996
		background Ω	0.06050	1.00000	0.06050	1.00000	
		foreground Ω	0.06050	1.00000	22.91392	22.91392	
**Dolphinω_dolphin_**	−1469.322896	Site class	0	1	2a	2b	
		proportion	0.89723	0.00523	0.00923	0.00005	
		background Ω	0.05851	1.00000	0.058 51	1.00000	
		foreground Ω	0.05851	1.00000	1.00000	1.00000	

To determine whether specific *Hox* genes have made parallel contributions to developmental changes in both the bat and the dolphin, and to avoid interference between these two lineages, we tested the levels of selective pressure when both lineages (bat common ancestor and dolphin) were set as foreground branches. In this analysis no statistical evidence was found to suggest that any of the *Hox* genes had undergone positive selection (Table S5).

### Survey for positive selection in newly sequencing data

A total of twenty-four complete *Hox* genes were successfully amplified (for both exons) from at least one bat species, with the coding sequences identified by comparison with reference sequences. Full-length coding sequence were obtained for: *Hoxa1, a2, a3, a6, a7, a9, a11, a13; Hoxb1, b2, b3, b5, b6, b7, b8, b9; Hoxc4, c5, c8, c9, c10, c11, c12; Hoxd10* (Table S2 lists each exon that was amplified for each species). Due to the limited size of our tissue samples for two cetacean species (fin whale and finless porpoise, Table S1), only six genes (*Hoxa2, Hoxb1, Hoxb8, Hoxb9, Hoxc6, Hoxd12*) were successfully amplified (see Table S2). To confirm the orthology of these genes, we used the predicted amino acid sequences of these genes to construct a ML tree with human and mouse gene sequences as references. Each *Hox* ortholog clustered into a monophyletic group with high bootstrap values (Figure S1).

The selective pressures acting on *Hox* genes were re-analyzed using newly sequenced genes, yielding results that validated the result from the genome analysis that most *Hox* genes from bats are evolving conservatively (Table S6). However, unlike the genomic analysis, analysis of the newly sequenced *Hox* genes found statistically significant evidence for positive selection in the *Hoxb2* gene along the bat ancestral branch (LRT test, 2⊿l = 4.4407, df = 1, *P*<0.05) with one positively selected site (226 E) having a BEB value greater than 0.95 in branch-site test 2 commonly existed in the newly sequenced bat data (Table 2). Of the six *Hox* genes amplified from the cetaceans *Neophocaena phoconoides* and *Balaenoptera physalus, Hoxb1* showed a marginal signal for positive selection (LRT test, 2⊿l = 3.7105, df = 1, *P* = 0.054) based on the branch-site models (Table S6). Since *Hoxd12* was suggested to have undergone positive selection in cetaceans by branch-site test 1 in a previous study [46], we combined the published sequences of this gene together with our new data and re-did analysis, however, we failed to find any significant evidence for positive selection using the rigorous branch-site test 2.

**Table 2:** The parameters of selective pressure for*Hoxb2* in bats from newly sequenced data.

Model	Inl			Estimates of parameters			
**MD**	−1077.467401						
**MI**	−1072.584702						
**Branch-Site Test 2**							
**The common ancestor of bats**	−1067.937132	Site class	0	1	2a	2b	Positively selected site
		proportion	0.909609	0.07522	0.01402	0.00116	226 E 0.963
		Background Ω	0.07047	1.00000	0.06050	1.00000	
		Foreground Ω	0.07047	1.00000	42.75951	42.75951	
**The common ancestor of batsω_bat_**	−1069.937132	Site class	0	1	2a	2b	
		proportion	0.79088	0.06581	0.13230	0.01101	
		Background Ω	0.06853	1.00000	0.068 53	1.00000	
		Foreground Ω	0.06853	1.00000	1.00000	1.00000	

## Discussion

Both Chiroptera and Cetacea have evolved specialized body plans and associated physiological systems for flying in the sky or swimming in water. The molecular mechanisms underlying these key morphological and physiological specializations have received increasing interest in recent years. *Hox* genes have long been recognized as extensively participating in major events during ontogeny (e.g. body plan, skeleton pattern, hindbrain segmentation, and limb form), primarily through variation in sequence or expression pattern [16]. Here, we systematically surveyed *Hox* protein sequences from genomic data during the origin of flight in Chiroptera and swimming in Cetacea.

The *Hox* gene sequences used in our genomic analyses comprise 39 genes in bats and 33 genes in dolphins, which represent all, or nearly all, of the family members. All members of the *Hox* gene family in the mammals we investigated experienced strong purifying selection (Figure 2(A), Table S5), suggesting that the general evolutionary pattern for mammalian Hox genes is very conservative. However, in bats and dolphins a different evolutionary pattern for the *Hox* genes was found. Using a relatively relaxed model (model M1) in PAML, it was found that several *Hox* genes in bats showed higher *dN/dS* ratios compared to dolphins (Figure 2(B)). These *Hox* genes in bats may have roles in their adaptation to flight. Since *Hoxa1* has a role in the segmentation of the hindbrain [28], it is possible that the changed *Hoxa1* gene of bats is a result of nature selection which would be beneficial in some kind of special characters, such as flight. Adaptation of *Hoxa10*, which promotes the regulation of hematopoietic lineage commitment [47], may have aided in oxygen transport in blood to help increase metabolism. Similarly, adaptation of *Hoxc8*, which has roles in enhancing the regulation of fat tissue types [27] and cartilage differentiation [48], may have adaptive roles in metabolism and the skeleton, while *Hoxd9* and *Hoxd12*, which are involved in forelimb development [49], may have a role in wing evolution. In contrast, only one gene, *Hoxc6*, which may regulate the promoter activity of neural cell adhesion molecule (N-CAM) gene [50], showed obviously higher *dN/dS* values in the dolphin.

However, when the rigorous branch models and branch-site models (test 2) with adjusting for false positive are considered, our results based on the mammalian genome sequences suggest that all of the *Hox* genes in bats have continued to evolve very conservatively. No genes were found to show significant evidence of positive selection. Since the genome of the flying fox bat (*P. vampyrus*) is of only draft quality (2.63X), and thus potentially contains errors, we newly sequenced and re-analyzed 24 *Hox* genes from an additional 23 bat species to amplify the breadth of this study and to validate our results based on the limited number of genome sequences. Results from this more extensive dataset, analyzed with branch-site models, are largely in accord with the genomic analysis, except that it revealed that *Hoxb2* showed significant evidence of positive selection in all of the successfully amplified bat samples, with the positively selected site (226) having a polarity change from Glu (negative charge) to Pro (non-polarity). This amino acid substitution may affect the function or structure of *Hoxb2*. *Hoxb2* has previously been shown to be associated with pancreatic cancer and participates in the migration of mouse pontine neurons [51], [52]. The pancreas is responsible for secreting digestive enzymes that break down carbohydrates, proteins, and lipids, thus the modified *Hoxb2* of bats may change the rate of enzyme secretion to enhance the digestion and absorption of nutrients to provide the additional energy required for flight. On the other hand, as genes in the *Hox* paralog group 2 control the neuronal fate within the hindbrain [53], positively selected *Hoxb2* may also play a special role during the hindbrain development. Thus, while our observations indicate that *Hoxb2* is a candidate gene, identifying which of its important diverse roles in the pancreas and neurons has adapted required additional functional experimentation.

A similar analysis of the dolphin genomic data suggested that only the *Hoxb9* had experienced positive selection in Cetaceans, with the change providing significance being at site 175, which changes from Pro (hydrophilic) to Val (hydrophobic). Mice bearing a homozygous deletion of *Hoxb9* have been shown to have an abnormal fusing pattern between the first and second ribs, resulting in a reduction of the thymic space in the upper thoracic region [54]. The first two ribs in the dolphin skeletal system in the thoracic cavity are also fully fused [55]. Since the timing and growth of ribs influences the development of the sternum, the abnormal separation and growth of the ribs likely results in the ribs joining the sternum unevenly and causing distortion [55]. With these changes the thymic region forms a flared cup-like recess, which could collapse under the pressure of a deep dive without being damaged in the dolphin [14]. The mutated *Hoxb9* may contribute to the special function of the rib pattern in the dolphin. Acquisition of impressive fore-flippers also required a series of reformed skeletal elements in the cetacean forelimbs, with all four *Hox9* genes (*Hoxa9*, *Hoxb9*, *Hoxc9*, and *Hoxd9*) acting in concert to establish the forelimb posterior domain by regulating *Hand2* expression in this region [56]. As *Hoxb9* has roles in the two above-mentioned skeletal characters of cetaceans, we speculate that the evolutionary changes in *Hoxb9* in the dolphin may be linked to changes in the rib pattern and forelimb development.

Previous research, using the branch-site test 1, concluded that *Hoxd12* underwent positive Darwinian selection [46], however it was not identified from the genomic data by the more rigorous branch-site test 2 in our analysis. To further confirm and examine this difference in results, we combined the previously published sequences with our new sequence data and re-did the analysis. Our re-analysis also failed to find a significant signal for positive selection in the *Hoxd12* gene using the rigorous branch-site test 2. A possible explanation for the conflicting observations is that since *Hox* genes are very important for development, then any short-term positive selection signal will be obscured by the extremely strong long-term purifying selection. This will mean that any change in the species, tree topologies, or even models used for the analyses of selective pressure likely influence the results. This property may also explain the conflicting observations of seen for *Hoxb2* between the genomic sequences (no signal for positive selection) and our newly sequencing data (signal for positive selection).

Our analysis of the *Hox* gene indicates that, like other terrestrial mammals, *Hox* genes are experiencing very strong purifying selection, thus it is very hard to detect significant signals for positive selection from the evolutionary pressure to explain the specialized development in Chiroptera and Cetacea. However, different selective patterns of *Hox* genes between these two groups can still be found from the results of both less strict models (model M1 (Table S5)) and rigorous models (branch models and branch-site test 2 by putting bats and dolphin together as foreground branches (Table S5)). Difference might be caused by distinct developmental innovations, since bats and cetaceans independently evolved. A conflict, however, appears to exist between the diverse phenotypes and conserved genotypes in both bats and dolphin. The *Hox* gene family is ancient and conserved, encoding transcription factors that are essential for the cis-regulation of the expression level of a series of downstream target genes [16]. These genes control the specification of regional identities along the anterior-to-posterior body axis and associated structures, either due to *Hox*-protein-function or *Hox*-expression-pattern [16]. In addition to mutation of the *Hox* protein sequence, which could induce functional changes, changes in *Hox* gene expression patterns have been highlighted as contributing to morphological evolution [16], [57]. Recent research has also highlighted the importance of timing of *Hox* gene expression (*Hoxa13* and *Hoxd13*), rather than the conserved gene structure, in marsupial limb development [58]. In addition, other genes, such as *Fgf8* [59] and *Shh* [60], show a similar requirement for their expression patterns during early development. Thus, examining the evolution of the upstream regulatory elements or the downstream target regions of *Hox* genes in Chiroptera and Cetacea may be another interesting project. Here, the conserved genotypes found in our study suggest that natural selection appears to prefer *Hox* genes with conserved coding regions but having diverse expression patterns, a pattern that could provide flexibility during the evolution of bats and cetaceans.

## Conclusions

Our findings suggest that signals of adaptive evolution could be detected for two genes (*Hoxb2* in bats, and *Hoxb9* in cetaceans), implying that these genes may have roles associated with the modifications of body plan or associated systems, or adaptations to these modifications, for the unique lifestyles of Chiroptera and Cetacea. Although there are differences in the selective patterns, when compared with terrestrial mammals, strong purifying selection still plays the most important role, such that the coding regions of most *Hox* genes are conserved in both Chiroptera and Cetacea. We speculate that the evolutionary pattern for *Hox* gene family varies for expression but is conservative for sequence in bats and cetaceans, which could induce the most flexibility and the least lethality. In this study, we characterized the pattern of adaptive evolution for the entire *Hox* gene family in Chiroptera and Cetacea from protein coding sequences. Additional functional experiments are required to characterize the specific contributions of the identified adaptive changes. In addition, our results have another important implication, that the deep genetic sampling of particular clade is a powerful method to confirm genomic results generated from a few species.

## Supporting Information

Figure S1 **A ML tree of newly sequenced** ***Hox*** **genes.** Topology of the ML tree is based on the amino acid sequences of all of the *Hox* genes generated by combining our newly sequenced gene sequences from bats and cetaceans with those from human and mouse. Numbers along the branches are bootstrap support values. Values below 70 are not shown.
(TIF)

Table S1 **Species used in this research.**
(DOCX)

Table S2 **Primers for amplifying** ***Hox*** **genes, and accession numbers for the newly sequencing data.** Primers used for amplifying *Hox* genes in Cetaceans are indicated in red. The exon 2 sequences for *Hox* genes (*Hoxc11* and *Hoxd12*) were less than 200 bases in length, and thus not submitted to GenBank, these sequences (labeled as short) are given in Table S3. The genes participated in analysis are indicated in green.
(XLSX)

Table S3 **Sequences of short exons that are less than 200** **bp from the newly sequencing data. This data could not be submitted to GenBank.**
(XLSX)

Table S4 **Ensembl transcript IDs and GenBank accession numbers of all** ***Hox*** **genes used in this study.**
(XLSX)

Table S5 **Analysis of the selective pressure acting on** ***Hox*** **genes derived from the genomic data.**
(XLSX)

Table S6 **Analysis of the selective pressure acting on** ***Hox*** **genes derived from the newly sequencing data.**
(XLSX)
