# Supplementary figures and images for: Adaptive Evolution of the Hox Gene Family for Development in Bats and Dolphins
Source: PLoS One. 2013 Jun 25;8(6):e65944. doi: 10.1371/journal.pone.0065944 (PMC3692524; doi:10.1371/journal.pone.0065944)

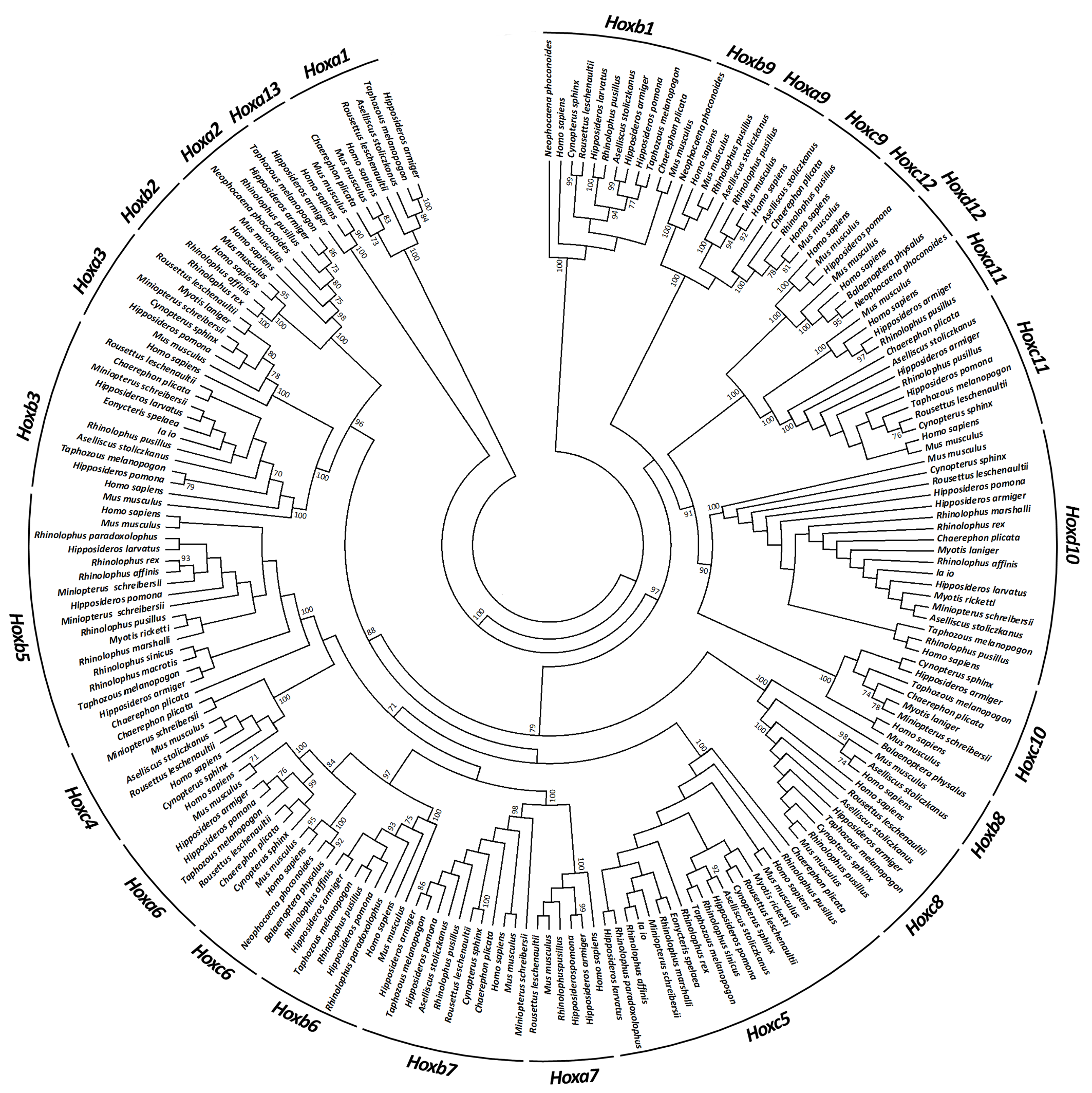

Supplement: Figure S1 — A ML tree of newly sequenced Hox genes. Topology of the ML tree is based on the amino acid sequences of all of the Hox genes generated by combining our newly sequenced gene sequences from bats and cetaceans with those from human and mouse. Numbers along the branches are bootstrap support values. Values below 70 are not shown. (TIF) [file pone.0065944.s001.tif]
