# Supplementary material for: Adaptive Evolution of the Hox Gene Family for Development in Bats and Dolphins
Source: PLoS One. 2013 Jun 25;8(6):e65944. doi: 10.1371/journal.pone.0065944 (PMC3692524; doi:10.1371/journal.pone.0065944)
Supplement: Table S1 — Species used in this research. (DOCX) [file pone.0065944.s002.docx]

**Table S1: Species used in this research.**

| **Order** | **Family** | **Genus** | **Species** | **Common Name** | **Locality** |
| --- | --- | --- | --- | --- | --- |
| Chiroptera | Emballonuridae | *Taphozous* | *Taphozous melanopogon* | Black-bearded Tomb Bat | Guangxi Province, China |
|  | Hipposideridae | *Aselliscus* | *Aselliscus stoliczkanus* | Stoliczka's Trident Bat | Yunnan Province, China |
|  | Hipposideridae | *Hipposideros* | *Hipposideros armiger* | Great Roundleaf Bat | Yunnan Province, China |
|  | Hipposideridae | *Hipposideros* | *Hipposideros larvatus* | Intermediate Roundleaf Bat | Guangxi Province, China |
|  | Hipposideridae | *Hipposideros* | *Hipposideros pomona* | Pomona Roundleaf Bat | Yunnan Province, China |
|  | Molossidae | *Chaerephon* | *Chaerephon plicata* | Wrinkle-lipped Free-tailed Bat | Yunnan Province, China |
|  | Rhinolophidae | *Rhinolophus* | *Rhinolophus affinis* | Intermediate Horseshoe Bat | Yunnan Province, China |
|  | Rhinolophidae | *Rhinolophus* | *Rhinolophus ferrumequinum* | Greater Horseshoe Bat | Yunnan Province, China |
|  | Rhinolophidae | *Rhinolophus* | *Rhinolophus macrotis* | Big-eared Horseshoe Bat | Yunnan Province, China |
|  | Rhinolophidae | *Rhinolophus* | *Rhinolophus marshalli* | Marshall's Horseshoe Bat | Yunnan Province, China |
|  | Rhinolophidae | *Rhinolophus* | *Rhinolophus paradoxolophus* | Bourret's Horseshoe Bat | Yunnan Province, China |
|  | Rhinolophidae | *Rhinolophus* | *Rhinolophus pusillus* | Least Horseshoe Bat | Yunnan Province, China |
|  | Rhinolophidae | *Rhinolophus* | *Rhinolophus rex* | King Horseshoe Bat | Yunnan Province, China |
|  | Rhinolophidae | *Rhinolophus* | *Rhinolophus sinicus* | Chinese Rufous Horseshoe Bat | Yunnan Province, China |
|  | Vespertilionidae | *Ia* | *Ia io* | Great Evening Bat | Yunnan Province, China |
|  | Vespertilionidae | *Miniopterus* | *Miniopterus schreibersii* | Common Bent-wing Bat | Yunnan Province, China |
|  | Vespertilionidae | *Myotis* | *Myotis laniger* | Chinese water myotis | Guangxi Province, China |
|  | Vespertilionidae | *Myotis* | *Myotis ricketti* | Rickett's Big-footed Bat | Guangxi Province, China |
|  | Vespertilionidae | *Nyctalus* | *Nyctalus noctula* | Common Noctule | Guangxi Province, China |
|  | Vespertilionidae | *Tylonycteris* | *Tylonycteris pachypus* | Lesser Bamboo Bat | Yunnan Province, China |
|  | Pteropodidae | *Cynopterus* | *Cynopterus sphinx* | Greater Short-nosed Fruit Bat | Yunnan Province, China |
|  | Pteropodidae | *Eonycteris* | *Eonycteris spelaea* | Cave Nectar Bat | Yunnan Province, China |
|  | Pteropodidae | *Rousettus* | *Rousettus leschenaultii* | Leschenault's Rousette | Yunnan Province, China |
| Cetacea | Balaenopteridae | *Balaenoptera* | *Balaenoptera physalus* | Fin Whale | Unclear |
|  | Phocoenidae | *Neophocaena* | *Neophocaena phoconoides* | Finless Porpoise | Hubei Province, China |
